# Unexpected differential metabolic responses of *Campylobacter jejuni* to the abundant presence of glutamate and fucose

**DOI:** 10.1007/s11306-018-1438-5

**Published:** 2018-10-23

**Authors:** Justin J. J. van der Hooft, Wejdan Alghefari, Eleanor Watson, Paul Everest, Fraser R. Morton, Karl E. V. Burgess, David G. E. Smith

**Affiliations:** 1 Bioinformatics Group, Wageningen University, 6708PB Wageningen, The Netherlands; 2 King Abdulaziz University, Jeddah, 21589 Kingdom of Saudi Arabia; 3 Institute of Biological Chemistry, Biophysics & Bioengineering, Heriot-Watt University, Edinburgh, EH14 4AS UK; 4 Moredun Research Institute, Pentlands Science Park, Bush Loan, Penicuik, EH26 0PZ UK; 5 School of Veterinary Medicine, University of Glasgow, Bearsden Road, Glasgow, G61 1QH UK; 6 Glasgow Polyomics, College of Medical, Veterinary and Life Sciences, University of Glasgow, Glasgow, G12 8QQ UK

**Keywords:** *Campylobacter jejuni*, Metabolomics, HILIC chromatography, Mass spectrometry fragmentation, Sulphur metabolism

## Abstract

**Introduction:**

*Campylobacter jejuni* is the leading cause of foodborne bacterial enteritis in humans, and yet little is known in regard to how genetic diversity and metabolic capabilities among isolates affect their metabolic phenotype and pathogenicity.

**Objectives:**

For instance, the *C. jejuni* 11168 strain can utilize both l-fucose and l-glutamate as a carbon source, which provides the strain with a competitive advantage in some environments and in this study we set out to assess the metabolic response of *C. jejuni* 11168 to the presence of l-fucose and l-glutamate in the growth medium.

**Methods:**

To achieve this, untargeted hydrophilic liquid chromatography coupled to mass spectrometry was used to obtain metabolite profiles of supernatant extracts obtained at three different time points up to 24 h.

**Results:**

This study identified both the depletion and the production and subsequent release of a multitude of expected and unexpected metabolites during the growth of *C. jejuni* 11168 under three different conditions. A large set of standards allowed identification of a number of metabolites. Further mass spectrometry fragmentation analysis allowed the additional annotation of substrate-specific metabolites. The results show that *C. jejuni* 11168 upon l-fucose addition indeed produces degradation products of the fucose pathway. Furthermore, methionine was faster depleted from the medium, consistent with previously-observed methionine auxotrophy.

**Conclusions:**

Moreover, a multitude of not previously annotated metabolites in *C. jejuni* were found to be increased specifically upon l-fucose addition. These metabolites may well play a role in the pathogenicity of this *C. jejuni* strain.

**Electronic supplementary material:**

The online version of this article (10.1007/s11306-018-1438-5) contains supplementary material, which is available to authorized users.

## Introduction

*Campylobacter jejuni* is a significant and relevant zoonotic pathogen as a frequent cause of debilitating intestinal infection. *C. jejuni* are the commonest causes of foodborne infections in most developed countries (Sheppard et al. 2012) and are also considered to be the commonest cause of enteric disease in children in developing countries (Platts-Mills and Kosek 2014). For instance, the recent intestinal infectious disease (IID2) survey in the UK estimates ~ 65,000 *Campylobacter* cases per annum resulting in a cost to the economy of approximately £900 million. Similar impact is recorded across European countries as well as US and Canada. The major reported sources of human *Campylobacter* infections are livestock animals in which these bacteria are widely distributed. Notably, poultry act as the main source of human infection although ruminants and pigs are also recognised as reservoirs of these zoonotic infections (Zorman et al. 2006; Hutchison et al. 2005; Hermans et al. 2011). Campylobacters are usually carried in animals in the absence of overt signs of disease, although exposure of humans typically results in enteric disease. *Campylobacter’s* metabolism has been associated with its pathogenicity through epidemiological and genomics studies (Zautner et al. 2011, 2012; de Haan et al. 2012).

*Campylobacter jejuni* and related species are near-ubiquitous residents of the intestinal tracts of mammalian and avian species and many studies show limited survivability outside these niches. It is widely reported that metabolic capabilities of *Campylobacter* species are restricted in comparison to other common intestinal tract residents (e.g. *Bacteroidetes* and *E. coli*) hence, *C. jejuni* and related species have been considered to be metabolically fastidious. Characterisation of metabolic capabilities of Campylobacters has mainly focussed on defining substrates utilised as energy and/or carbon sources and on associated proteins/genes. Notably, *C. jejuni* and *Campylobacter coli* are typically non-glycolytic, lacking both necessary sugar transport systems and several enzymes involved in glycolysis (Hofreuter 2014; Wagley et al. 2014). Rather, these bacteria utilise several TCA cycle intermediaries (pyruvate; malate; succinate; fumarate), some organic acids (acetate; lactate; hydroxylbutyrate; formate) and several amino acids as substrates (Mohammed et al. 2004; Stahl et al. 2012; Wright et al. 2009), and *C. jejuni* strains were observed to be methionine auxotrophic (Alazzam et al. 2011; Tenover and Patton 1987). Several investigations have identified aspartic acid, glutamic acid, proline and serine as primary amino acid substrates (Wagley et al. 2014; Wright et al. 2009) for many strains whilst selected strains of *C. jejuni* possess determinants which permit utilisation of additional amino acids, principally asparagine and glutamine, and oligopeptides like γ-glutamyl-cysteine and glutathione (Hofreuter et al. 2008). Also, although not utilised as a carbon source, cysteine has recently been identified as a crucial source of sulphur (Vorwerk et al. 2014).

Furthermore, despite being classified as asaccharolytic, some *Campylobacter* strains can utilise fucose (Muraoka and Zhang 2011; Stahl et al. 2011) although the utilisation pathway for fucose has not been entirely specified in *Campylobacter*. Fucose is a common mucin component and chemoattractant (Muraoka and Zhang 2011; Wisessombat et al. 2010; Tu et al. 2008); which influences gene expression and an advantage in neonatal piglet intestinal colonisation by fucose use was reported (Muraoka and Zhang 2011; Stahl et al. 2011). Mucin is a major component of mucus—it can be regarded as the interface of the gastrointestinal mucosa with micro-organisms and it provides viscous consistency (Freitas and Cayuela 2000). Fucose can be present in the gut as a breakdown product of mucus where it is produced and released by other gut colonizers. Transcriptomics analysis showed large changes in transcript abundancies upon presence of fucose with 74 transcripts up-regulated and another 52 down-regulating (Stahl et al. 2011), suggesting a broader impact than merely activating the fucose breakdown pathway. Significantly, the capacity of *C. jejuni* strains to utilise “accessory” substrates such as fucose has been shown to correlate with colonisation and pathogenicity in avian and mammalian species. For instance, the ability to utilise glutamine has been shown to provide an advantage in colonisation of chicken and mouse intestinal tracts (Hofreuter et al. 2008; Barnes et al. 2007).

Glutamic acid is one of the four preferential amino acids of *Campylobacter* (Wagley et al. 2014; Hofreuter et al. 2008; Guccione et al. 2008) and its presence in the gut is mainly from a dietary source, in particular processed food. We therefore postulate that the influence of the nutritional milieu in each of these host species and the adaptation of *C. jejuni* to the conditions prevailing in each of these different intestinal environments is of relevance to infectivity and pathogenicity.

Investigations into the metabolic capabilities of *C. jejuni* and establishing how these contribute to niche occupation, host species colonisation and virulence have recently begun (Howlett et al. 2014) to both improve understanding and offer options towards disease control of *Campylobacter* spp. Hence, gaining a clearer understanding of metabolism of these pathogens and the influence of different substrates on their physiology is important in defining adaptation and colonisation in the different host niches as well as—potentially—pathogenicity. To date, there have been few systematic evaluations of the influence of carbon/energy sources on *C. jejuni* molecular phenotype. “Global” metabolomics characterisation using high resolution mass spectrometry is an increasingly useful approach for characterising metabolism (Gao and Xu 2015; Xie et al. 2013) although to date few studies have taken advantage of untargeted metabolomics approaches to study *Campylobacter* strains (Howlett et al. 2014) to gain greater understanding of *C. jejuni* adaptation to availability of substrates.

Herein we present a preliminary survey of the extracellular metabolome in *C. jejuni* strain 11168, a widely utilised reference strain for which a fully annotated genome sequence is available (Parkhill et al. 2000)—provided with either excess amounts of l-glutamic acid or l-fucose in growth medium. Detailed analysis of *Campylobacter*’s metabolic response has not previously been obtained through metabolomics and here we report the profile of substrates utilised, key features of the *C. jejuni* metabolome, and differential metabolome profiles upon supplementation of glutamate and fucose. For this analysis, we employed an untargeted hydrophilic liquid chromatography coupled to mass spectrometry (pHILIC-MS) approach focusing on small polar metabolites (i.e., m/z ≤ 400 Da), enriched by pHILIC-MS fragmentation experiments to gain additional structural information on detected metabolites.

The approach utilised herein provided 101 metabolite assignments across the three media compositions and three time points of metabolites that (i) serve as energy substrates, (ii) are part of the core extracellular metabolome of *C. jejuni*, or (iii) are novel metabolites released under specific conditions. Of those metabolites, 23 compounds were verifiable through comparison with standards. Among the 78 compounds for which no standard was present, 39 could be confidently matched to reference MS/MS data or one candidate structure was most likely based on diagnostic evidence [Metabolite Standards Initiative Metabolite Identification (MSI MI) level 2] (Sumner et al. 2007). Our analysis showed utilisation of core and accessory components as energy source by *C. jejuni*, major changes to extracellular metabolome upon relative minor changes to the media conditions caused by the production and secretion of novel *C. jejuni* metabolites (see Fig. 5). To date, this is the most extensive survey of the *C. jejuni* extracellular metabolome in response to different medium conditions. The study provides novel insight into the metabolic capabilities of this important zoonotic pathogen as well as new avenues to investigate environmental adaptability and disease control for this organism.

## Materials and methods

### Bacterial strains and culture preparation

*Campylobacter jejuni* strain 11168O (original isolate) was used in this investigation. This strain is a key reference strains used in investigations of many aspects of *C. jejuni* biology. A fully annotated and updated genome sequence has been available for variants of this strain since 2000 (Parkhill et al. 2000) and was updated in 2007 (Gundogdu et al. 2007). Bacteria were stored as glycerol stocks at − 80 °C and were recovered on Skirrow’s Agar (E&O Laboratories, Bonnybridge, UK). For metabolome preparation, cultures were plated onto replicate Skirrow’s Agar plates and cultured for 48 h at 37 °C in an atmosphere comprising 5% O_2_, 5% CO_2_, 2% H_2_, 88% N_2_ maintained in an A35 Anaerobic Workstation (Don Whitley Scientific Limited, Shipley, UK). Triplicate media preparations were prepared for each of the three media permutations: MEMα (Catalogue No. 41061; Life Technologies); MEMα plus 20 mM glutamic acid (MEMαG) and MEMα plus 25 mM fucose (MEMαF). All media were additionally supplemented with 20 µM iron(II) sulphate heptahydrate. *C. jejuni* colonies were recovered from plates using adsorbent swabs and suspended in prepared media; absorbance (600 nm) of bacterial suspensions was measured and adjusted to 0.01. Then, 5 ml of eac broth culture were then transferred to vented Corning® T25 tissue culture flasks and flasks were incubated horizontally to provide for largest possible surface area. Culture was carried out in an A35 anaerobic chamber (Don Whitley Scientific) with a gas mixture of 5% oxygen:5% carbon dioxide:2% hydrogen:88% nitrogen (BOC). These growth conditions provided highly reproducible growth of *C. jejuni* 11168, consistently achieving approx. 10^8^ cfu ml^−1^ at the sampling timepoints of 4 h, and ≥ 10^9^ at 9 h and 24 h in all media formulations. Cfu was a much more reliable means of standardisation of growth than A600 which was 0.36, 0.69 and 0.59 for MEMα; MEMαG or MEMαF respectively at 9 h with cell yield approx. 10^9.5^. Similarly, at 24 h, A600 values were 0.54, 1.25 and 0.76 respectively whilst cfu ml^−1^ were calculated at 10^9.4^, 10^9.7^, 10^9.6^.

### Sample preparation

At the selected sampling times. 1 ml of culture was removed from each of the three replicate cultures for each media formulation, placed into a 1.5 ml Eppendorf tube on ice and immediately centrifuged (10,000×*g*, 5 min) at 4 °C. The upper portion (100 µl) of the supernatant was dispensed into 400 µl of extraction solvent (chloroform:methanol 1:3) and the sample was placed on ice for 30 min with occasional shaking. Samples were then centrifuged (15,000×*g*, 3 min, 4 °C) and the upper 200 µl of each sample was removed and stored at − 80 °C until analysed by LC–MS.

### Analytical instrumentation and settings

#### Chromatography

The samples were analysed using a Thermo Scientific Ultimate 3000 RSLCnano system (Thermo Scientific, CA, USA). The pHILIC separation was performed with a SeQuant ZIC-pHILIC column (150 × 4.6 mm, 5 µm) equipped with the corresponding pre-column (Merck KGaA, Darmstadt, Germany)—the column temperature was maintained at 25 °C. A linear biphasic LC gradient was conducted from 80% B to 20% B over 15 min, followed by a 2 min wash with 5% B, and 8 min re-equilibration with 80% B, where solvent B is acetonitrile and solvent A is 20 mM ammonium carbonate in water. The flow rate was 300 µl/min, column temperature was held at 25 °C, injection volume was 10 µl, and samples were maintained at 4 °C in the autosampler (Creek et al. 2011).

#### Mass spectrometry

The LC system was coupled to a Thermo Scientific Q-Exactive Orbitrap mass spectrometer equipped with a HESI II interface (Thermo Scientific, Hemel Hempstead, UK). The set up was calibrated (Thermo calmix) in both ionization modes and tuned for the lower m/z range before analysis. Full scan (MS1) data was acquired in positive and negative switching mode in profile mode at 35,000 resolution (at m/z 200) using 1 microscan, an AGC target of 10^6^ cts, a maximum injection time of 250 ms, with spray voltages + 3.8 and − 3.0 kV, capillary temperature 320 °C, heater temperature of 150 °C, sheath gas flow rate 40 a.u., auxiliary gas flow rate 5 a.u., sweep gas flow rate 5 a.u., a full scan mass window of 70–1050 m/z, and using m/z 74.0964 (+) and m/z 112.98563 (−) as locking masses.

#### Mass spectrometry fragmentation

Fragmentation data (LC–MS/MS) of selected samples was obtained in positive and negative ionization combined and separate fragmentation modes as described in (van der Hooft et al. 2016a). Briefly, for separate mode, a duty cycle consisted of one full scan (MS1) event and one Top5 (or Top10) MS/MS (MS2) fragmentation event, with full scan (MS1) resolution (at m/z 200) was set to 70,000, the AGC target set to 1 × 10^6^, and the maximum injection time set to 120 ms. MS/MS (MS2) resolution (at m/z 200) was set to 17,500, the AGC target set to 2 × 10^5^, MS/MS maximum injection time was set to 80 ms and the underfill ratio was set to 10%, with a resulting intensity threshold of 2.5 × 10^5^ cts. For combined mode, a duty cycle consisted of two of the above events in positive and negative ionization mode with the following modifications: full scan (MS1) resolution (at m/z 200) was set to 35,000, MS/MS resolution was set to 35,000, the AGC target was set to 1 × 10^5^, and the maximum MS/MS filling time was set to 120 ms with an underfill ratio of 20%, resulting in an intensity threshold of 1.7 × 10^5^. Further settings were as specified for full scan analysis above.

#### Data acquisition

Blank runs, quality control samples (beer and serum extracts) in accordance with standard procedures at Glasgow Polyomics to assess the performance of the mass spectrometer in terms of chromatography and mass intensities, and three standard mixes containing 150 reference compounds available from Glasgow Polyomics were run to assess the quality of the mass spectrometer and to aid in metabolite annotation and identification (Creek et al. 2011). Pooled sample containing all samples was run prior to and across the batch every 6th sample to monitor the stability and quality of the LC–MS run, whereas the samples were run in a randomized order. Thermo Xcalibur Tune software (version 2.5) was used for instrument control and data acquisition.

Immediately after acquisition, all raw files were converted into mzXML format, thereby centroiding the mass spectra and separating positive and negative ionization mode spectra into two different mzXML files using the command line version of MSconvert (ProteoWizard). Data quality assurance was performed and accurate masses of standards were obtained well within 3 ppm accuracy and intensities, pooled injections showed low variability, and intensities of the quality control samples (a beer extract and a serum extract) were within specifications.

#### Data processing and analysis

MzXML files were uploaded into IDEOM (Creek et al. 2012) and the substrate analysis was performed using the resulting peak list from which identified (matched) metabolites were selected. MzXML files were uploaded into PiMP (Gloaguen et al. 2017) that relies on XCMS (Smith et al. 2006) and MzMatch (Scheltema et al. 2011) for LC–MS peak picking and alignment across samples. Principal component analysis and comparative analysis were performed within PiMP. Substrate analysis was performed using the identified amino acids and other metabolites present in standard mixes run along the samples. Suspect analysis was performed by searching for expected m/z masses in the MS1 peak list (within 3 ppm) and subsequent interpretation of retention time and MS/MS spectra, if available, or based on the presence of the metabolite feature in a characterized cluster (e.g., histidine related). Fold changes and Log^2^ fold changes were determined for comparisons across different conditions and different time points—maximum and minimum Log^2^ fold changes were cut off at 13 and − 13, respectively, as they represent complete absence of a metabolite feature in one sample and higher/lower values would render graphs unreadable and the analytical set up is not able to measure higher fold changes accurately within its dynamic range. Selection criteria for untargeted metabolite features for metabolite annotation/identification are given in Table 1.

**Table 1:** Feature selection from untargeted analysis for metabolite identification and annotation

Comparisons	Log^2^ FC	Intensity	# Features
24 h Fuc/4 h Fuc and 24 h Fuc/24 h Med	≥ 6	≥ 2E6 24 h Fuc	25
24 h Fuc/4 h Fuc and 24 h Fuc/24 h Med	≤ − 1.5	≥ 2E6 4 h Med	7
24 h Glu/4 h Glu and 24 h Glu/24 h Med	≥ 1.5	≥ 5E6 24 h Glu	23
24 h Glu/4 h Glu and 24 h Glu/24 h Med	≤ − 1.5	≥ 2E6 4 h Med	9
24 h Med/4 h Med	≥ 2	≥ 2E6 24 h Med	37
4 h Med/24 h Med	≤ − 2	≥ 2E7 4 h Med	13

#### Metabolite annotation

Metabolite annotations are reported according to the Metabolomics Standards Initiative (MSI) Metabolite Identification (MI) levels: (1) for unambiguously identified, (2) for a spectral or literature match, (3) for a metabolite classification (e.g., based on Molecular Networking), (4) for metabolites that can be characterized by a retention time, mass, and fragmentation spectra if available (Sumner et al. 2007). Metabolites that matched to a standard and which fragmentation spectrum matched with that of reference MS/MS spectra were considered identified (MSI MI Level 1). If no such match was found, fragmentation spectra were analysed and compared to reference spectral databases [i.e., MzCloud (http://www.mzcloud.org) and Metlin (metlin.scripps.edu) (Tautenhahn et al. 2012)] and analysed using MaGMa (http://www.emetabolomics.org, Ridder et al. 2013).

#### Data availability

All MzXML files used for this study were uploaded into the GNPS repository (Wang et al. 2016) and can be accessed through here: ftp://massive.ucsd.edu/MSV000081290. The data set consists of both full scan and fragmentation data folders with each of them containing positive ionization mode and negative ionization mode data folders.

## Results

### Metabolic responses to added fucose and glutamic acid

Initial assessment of the data after processing through PiMP using principal component analysis (PCA; Supplementary Figure S-1) indicated that biological replicates clustered very closely together and, hence, were highly reproducible. PCA analysis also showed that *C. jejuni* metabolome composition was measurably distinct in each growth medium (including at time 0) and that composition changed over the course of bacterial growth. Note the substantial compositional differences from the 9 to 24 h time points indicating a shift in *Campylobacter’s* metabolism in all three conditions. Based on these initial results, the substrate use and the changes in excreted metabolites after 4 h and 24 h in medium only, l-glutamate addition, and fucose addition were investigated in more detail.

### Substrate depletion by *Campylobacter jejuni* under different conditions

*Campylobacter jejuni* is considered to be a micro-organism with restricted metabolic capabilities, with TCA cycle intermediaries and certain amino acids being recognised as common carbon and energy sources. There are many reports of the utilisation of serine, proline, glutamate and aspartate—along with several TCA cycle intermediates (e.g. pyruvate) as primary carbon and energy sources across *C. jejuni* strains (including strain 11168) (Mohammed et al. 2004; Stahl et al. 2012; Line et al. 2010). In addition to utilisation of common substrates, *C. jejuni* exhibits strain-dependent metabolic heterogeneity with some strains possessing genomic loci conferring utilisation of additional substrates such as glutamine, asparagine or fucose. Among these, *C. jejuni* strain 11168 possesses the genomic locus required for fucose uptake and utilisation (cj0485-cj0490) but not *ggt* (γ-glutamyl-transferase) or *ansB*s (periplasmic asparaginase) required for Gln and Asn utilisation, respectively (de Haan et al. 2012). Moreover, it has recently been reported that cysteine represents a crucial source of sulphur for *C. jejuni* (Vorwerk et al. 2014).

**Fig. 1:**
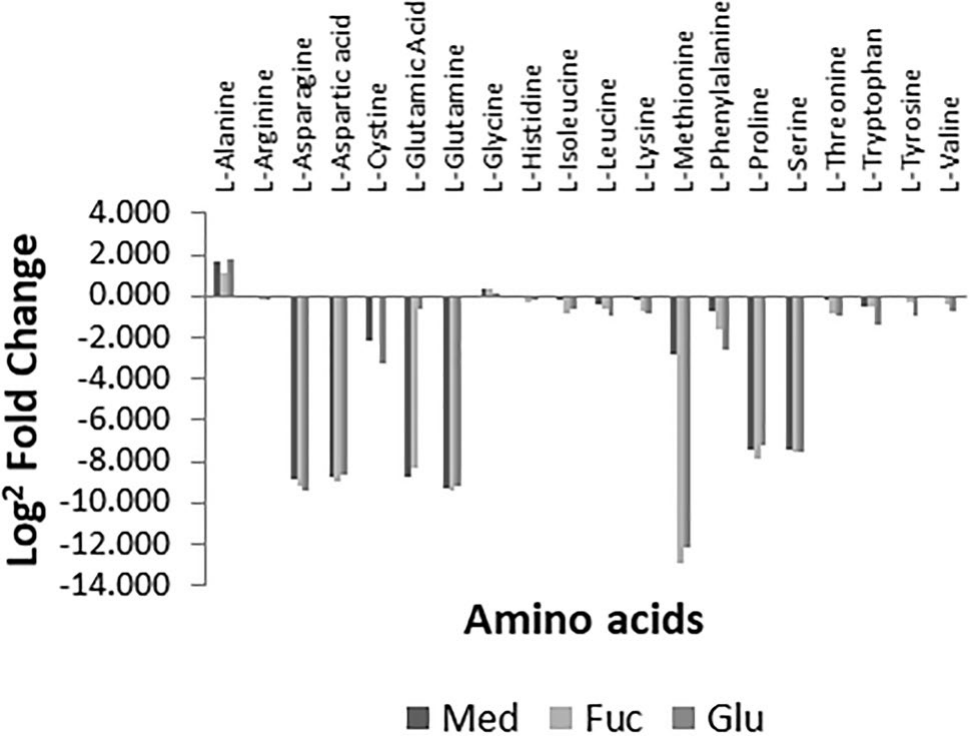
Amino acid analysis using IDEOM identifications to determine Log^2^ fold changes (mean of triplicate measurements) of amino acid peaks of 24 h compared with 0 h samples for medium only (Med), medium supplemented with fucose (Fuc), and medium supplemented with glutamic acid (Glu). It can be seen that several amino acids are preferably depleted from the medium. Further inspection revealed that aspartic acid, glutamine, methionine, proline, and serine are all completely depleted from the medium within 4 h. Glutamate was depleted under all conditions; in the glutamate-supplemented condition the Log^2^ fold change is less pronounced due to the glutamate surplus abundance

Primary amino acid substrates utilised by *C. jejuni* are components of the basal medium selected for this study (Supplementary Table 1) and we indeed observe depletion of *Campylobacter*’s preferred amino acid substrates Ser, Pro, Asp, and Glu (Wagley et al. 2014; Guccione et al. 2008) under all conditions (Fig. 1); with a number of those completely depleted within 4 h after inoculation. Fucose was also found to be depleted in a time-dependent manner in the fucose-supplemented medium—indicating use of the locus for fucose uptake and utilisation (discussed further below). In addition, our results show that this strain also depletes Asn, Gln, Cys, Met and Phe from the medium under all conditions. Surprisingly, both Asn and Gln were found to be depleted under all conditions despite *C. jejuni* strain 11168 not having the reported genomic loci for their uptake and use. Amongst the additional medium constituents, ascorbic acid and nicotinamide are completely depleted under all conditions (Supplementary Figure S-3); and pantothenate was also depleted from the medium—an observation not previously reported. Finally, we did observe an increase in the extracellular alanine (Fig. 1); which could be a by-product of energy metabolism; however, we cannot discriminate between l- and d-alanine in our global approach.

### Core extracellular *Campylobacter jejuni* metabolome

**Fig. 2:**
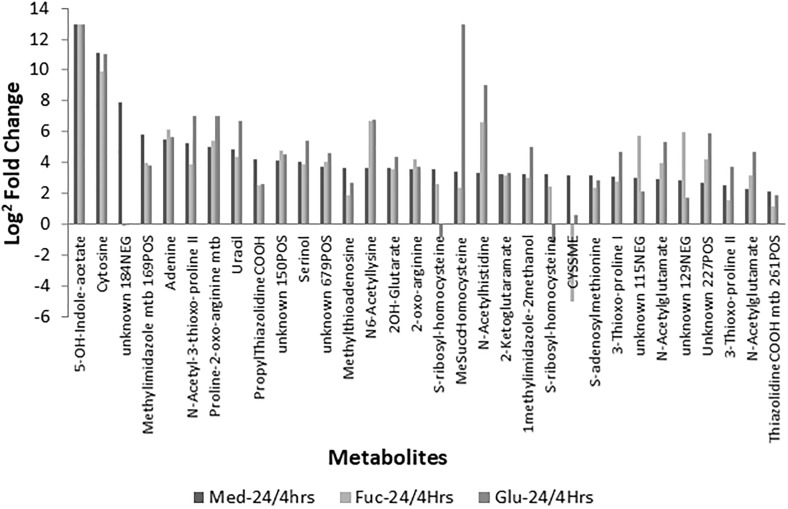
Upon growth in MEMα medium *Campylobacter jejuni* excreted a unexpectedly high number of metabolites into the medium. Plotted are the Log^2^ fold changes of selected metabolites based on 24/4 h non-supplemented MEMα comparison—for all the three conditions Med; non-supplemented MEMα (left), Fuc; fucose-supplemented MEMα (middle), Glu; glutamate-supplemented MEMα (right). It can be observed that most of the metabolite changes occurring upon growth in MEMα medium are similar across the two treatments and control and form a ‘base metabolic response’; however there are some exceptions. Some metabolites were detected in both negative and positive mode; hence their double presence here

*Campylobacter jejuni* excreted an unexpectedly high number of metabolites into the medium under all conditions. Given the high number of features [unique mass/charge-retention time (m/z-RT) pairs] that were found to be released by *C. jejuni* over the time course sampled, we set out to annotate a selected subset of those features (see Methods section) which resulted in 32 metabolite annotations (Fig. 2, Supplementary Table S-2). As can be observed, most of those metabolites are part of a common set of metabolites released into medium upon *C. jejuni* growth, independent of the conditions tested here. Amongst those, a number of cysteine-related metabolites were found such as the LuxS signalling pathway related *S*-adenosylmethionine and *S*-ribosyl-homocysteine (Plummer 2012; Vendeville et al. 2005). In addition, several purine metabolites, for example adenine and uracil, were increasingly present in the extracellular media. Nucleotides like adenosine have been reported as potential signalling molecules as well as small molecules involved in host–microbe interactions (Idzko et al. 2014). The metabolite with the greatest fold change we observed was 5-hydroxyindoleacetate (or an hydroxyindoleacatate isomer), a tryptophan metabolite, with a potential signalling role or host interaction like many other indole-based metabolites (Berstad et al. 2015) (see also Supplementary Information Sect. 1). There were some noticeable exceptions of core metabolites that were not or to a lesser extent released into the medium: e.g., *S*-(2-hydroxyethylmercapto)-cysteine (CYSSME), not commonly reported as being produced by bacteria. Furthermore, *S*-methylsuccinyl-homocysteine was released in higher abundancies by *C. jejuni* under excess glutamic acid.

A common set of metabolites was depleted from medium as found by comparing 4 h and 24 h non-supplemented MEMα samples of which we were able to annotate a subset of 8 (Supplementary Figure S-4). Again, we note depletion of methionine, notably its use by *C. jejuni* increased by the presence of both Glutamic acid and fucose. ThiomorphilineCOOH; however, is used less upon the presence of excess glutamic acid and fucose.

### Excess of glutamic acid presence alters metabolism of *Campylobacter jejuni*

**Fig. 3:**
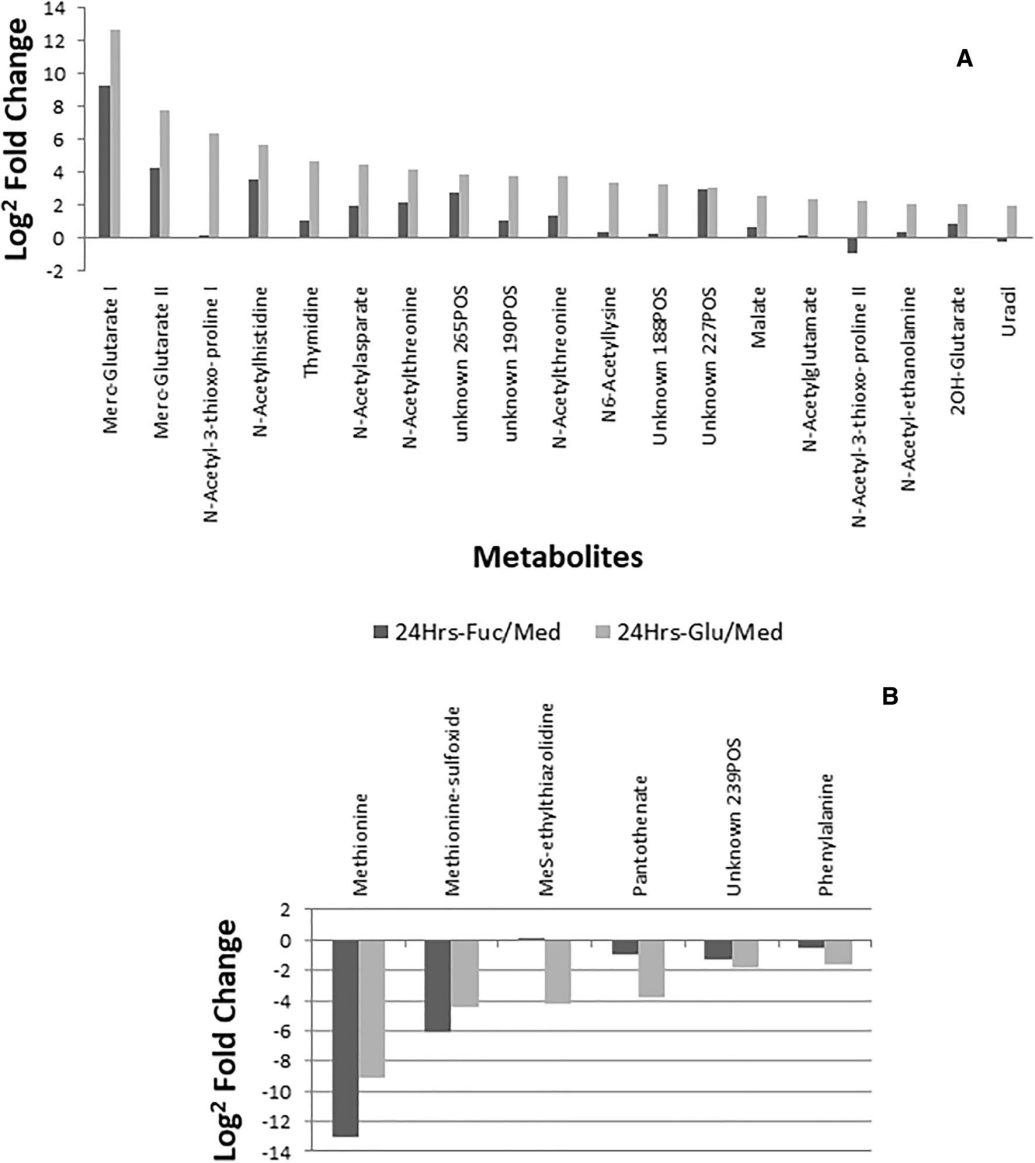
Most-differential metabolomic changes upon glutamate supplementation of selected metabolites based on 24/4 h fucose-supplemented and 24 h fucose-supplemented/24 h medium only comparisons. It can be observed that a wide range of acylated amino acids is excreted into the medium. Some metabolites were detected in both negative and positive ionization mode; hence their double presence here

The presence of excess glutamic acid (20 mM compared to 0.51 mM in unsupplemented medium) substantially changed the extracellular metabolome of *C. jejuni* with an (increased) presence of acetylated species (Supplementary Table S-2). As evident from Fig. 3, differential metabolites that are more abundantly released in the presence of excess glutamic acid are also increased in the presence of excess fucose. Some metabolites—*N*-acetylasparate and *N*-acetylthreonine, for example—are also released in absence of supplementation, although to a lesser extent. Again, there are several exceptions: Thymidine, *N*-acetyl-3-thioxo-proline, *N*6-acetyllysine, *N*-acetylglutamine, malate, uracil, and *N*-acetyl-ethanolamine are released in higher abundancies upon glutamic acid supplementation than fucose supplementation (Fig. 3). Thus, it seems likely that an excess of glutamic acid activates the acetylation of proteins, peptides and/or amino acids (Hu et al. 2010).

*Campylobacter* metabolizes glutamic acid under microaerobic conditions through the aspartate pathway with alpha-ketoglutarate (2-oxoglutarate) as intermediate and acetate as one of the products (Stahl et al. 2012; Guccione et al. 2008; Buckel and Barker 1974; Del Leon-Kempis et al. 2006; Laanbroek et al. 1978). We do observe increased release of 2-hydroxyglutarate, a related metabolite to alpha-ketoglutarate (Fig. 3). Acetate is too small to be observed with the methodologies used in this study and it can be used as substrate by *Campylobacter* as well, but we could expect an increase in acetate due to increased availability and turnover of glutamic acid. Thus, another explanation for the increased acetylated amino acids might be the increased available acetate pool that activates acetylation enzymes or results in chemical addition of the acetyl group to available molecules. However, we observe numerous metabolites released into the environment in higher abundancies upon glutamic acid addition (see also Supplementary Sect. 2) including the acetylated amino acids which may influence the virulence and metabolism of *C. jejuni*.

### Fucose presence induces production of novel *Campylobacter* metabolites

#### Differential metabolites in fucose added extracellular metabolome

**Fig. 4:**
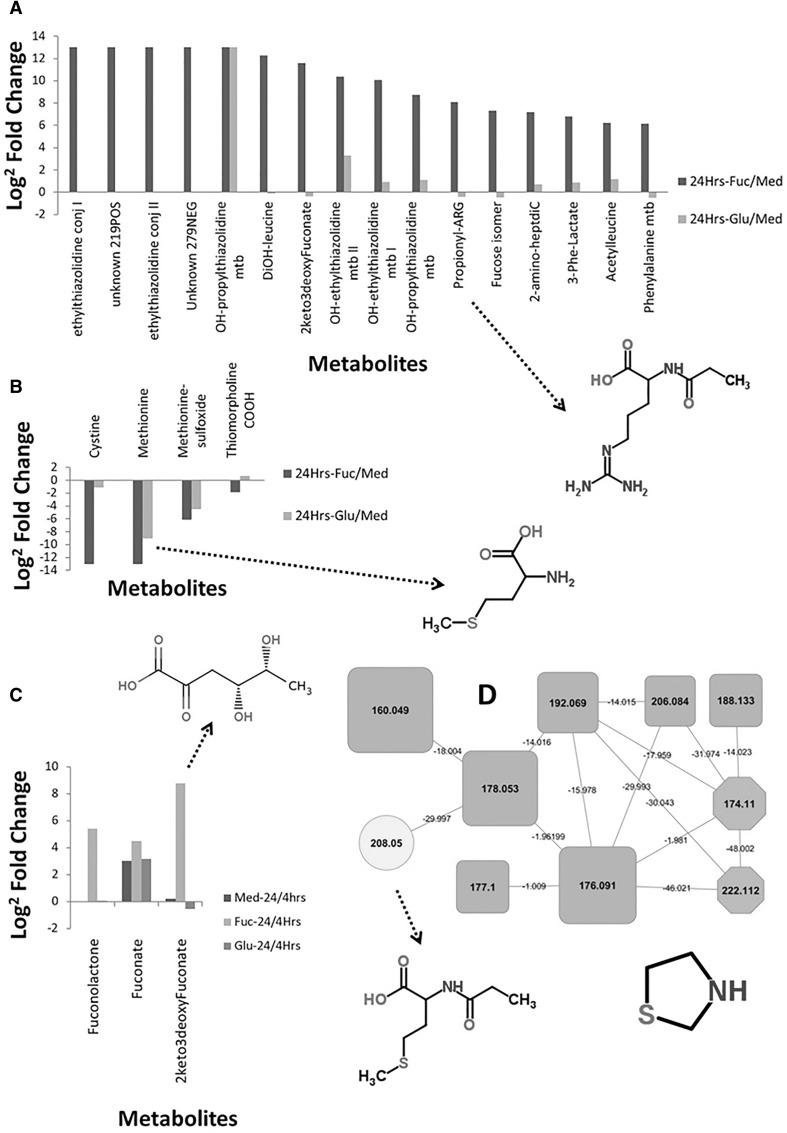
Most-differential metabolomic changes upon fucose supplementation of selected metabolites based on 24/4 h fucose-supplemented and 24 h fucose-supplemented/24 h medium only comparisons with metabolites **a** released into and **b** depleted from the medium. **c** Breakdown products of fucose were also found to be excreted in the medium by *Campylobacter*, thereby supporting the proposal of Stahl et al. that *Campylobacter jejuni* possesses a *Xanthomonas campestris*-like fucose utilisation pathway (Stahl et al. 2011). **d** Molecular networking provided additional evidence for metabolites detected only upon fucose supplementation of the medium. A number of annotated metabolites were thiazole-containing (the heteroatomic ring depicted). Arrows point towards examples of detected structures

Fucose addition to the growth medium caused substantial changes in the extracellular metabolome of *C. jejuni* at all three timepoints (4, 9, and 24 h after inoculation)—more so than glutamic acid supplementation based on the PCA (see Supplementary Figure S-1). Indeed, when investigating the most differential features in the extracellular metabolome upon fucose supplementation (Fig. 4a, Supplementary Table S-2), we can observe that all but one metabolite are specific for fucose supplementation. Further inspection revealed that the metabolite that also appeared to occur upon glutamic acid supplementation, hydroxypropyl-thiazolidine-4-carboxilic acid, was detected in both positive and negative mode ionization and in negative mode the 24 h glutamic acid samples contained minute amounts of a metabolite similar in mass (whereas nothing appeared after 4 or 9 h). In fact, the positive mode detection better reflects the differential secretion of this metabolite (see also Supplementary Table 2). Amongst the fucose-dependent metabolites, we observe thiazolidine-containing metabolites, leucine metabolites, and *N*2-propionylarginine. The thiazolidine ring structure is present in many biological active structures such as yersiniabactin, a siderophore, and thiocillins, an antibiotic family, thus its increased presence in the extracellular metabolome could indicate the activation of metabolic pathways generating bioactive factors.

We observed strong depletion of cystine, methionine, methionine-sulfoxide, and thiomorpholinecarboxylic acid, with the latter two presumably being chemically-formed products of methionine in the medium as they do not form part of the original growth medium mix. These are all sulphur-containing metabolites, which fits in well with the observation of sulphur-containing thiazolidines (cysteine metabolites) that are formed upon fucose supplementation. This observation also fits with previous studies showing Campylobacters to be methionine auxotrophic (Alazzam et al. 2011; Tenover and Patton 1987).

#### Metabolic evidence for fucose breakdown pathway in Campylobacter

The ability of certain *C. jejuni* strains to utilise fucose has been described although evidence for cognate enzymes and reaction intermediaries has been incomplete. Through a combination of the untargeted approach and suspect analysis, several compounds corresponding to fucose metabolism could be annotated (Supplementary Table S-2 and Supplementary Sect. 3). For example, 2-keto-3-deoxy-l-fuconate was picked up by the untargeted approach. Further inspection revealed two isomers appeared, with l-fuconolactone showing a number of fragments similar to δ-gluconic acid-δ-lactone (the glucose analogue of Fuconolactone which has MS/MS data in MzCloud) enabling its structural annotation. Similarly, fuconate was annotated using fragmentation similarities to Gluconate (see Supplementary Sect. 3 and Supplementary Figure S-5). Finally, pyruvate is one of the final products of the proposed fucose breakdown pathway. We do observe that pyruvate is depleted less from the medium under fucose supplementation (see Supplementary Figure S3); however, a possible explanation could be the replenishing of the pyruvate pool with products from fucose breakdown. Detection of these intermediates provides supporting evidence that fucose metabolism in *C. jejuni* closely functionally resembles that of *Xanthomonas campestris* as postulated by Stahl et al. (2011). Comparison of predicted functional motifs of fucose locus proteins sequences from *X. campestris* and *C. jejuni* identify four potential functional orthologues including the permease (Supplementary Table S-2) involved in fucose metabolism leaving three enzymes unidentified despite metabolic evidence.

#### Chemical alterations of medium upon addition of fucose

Finally, based on observations in the PCA (Supplementary Figure S-1) where the 4 h time points are relatively distant from each other, we inspected the 0 h control media samples. It is noteworthy that the supplementation of medium with either glutamic acid or fucose resulted in more additional metabolite features than expected; this was more pronounced in the fucose-supplemented medium suggesting spontaneous formation of adducts in the absence of bacteria. Fragmentation data obtained from control media and culture supernatants were used to create a molecular network in order to inspect if particular sets of compounds could be responsible for the alterations upon fucose addition. We observed a cluster of compounds present only at 0 h with the fucose supplementation—these slowly decreased in abundance over time (Supplementary Sect. 3 and Supplementary Figure S-6). We could annotate fucose conjugates to ten different amino acids (Supplementary Table S2). Using the molecular network, we also annotated additional metabolites belonging to the Thiazole and Histidine clusters at later timepoints leading to the annotation of Propionylmethionine which is released after 9 and 24 h into the extracellular metabolome with fucose supplementation. Figure 4 also shows Propionylarginine was released in these conditions. However, further analysis is warranted to investigate if this is generated by *C. jejuni* or a chemical product of these (preferred) amino acids and propionic acid exported by *C. jejuni*.

Thus, we not only observed that fucose was used as additional carbon source by *Campylobacter* when available as main accessory substrate, but its presence also reprograms *Campylobacter’s* metabolism more widely resulting in the release of novel *C. jejuni* metabolites. Other *omics* studies also revealed substantial changes in transcriptome and proteome—further evidence that the addition of fucose has a significant impact on *Campylobacter’s* phenotype (Stahl et al. 2011; Alghafari 2016).

## Discussion and conclusions

*Campylobacter jejuni* is one of the major causes of food-borne infections. A better understanding of the factors that influence its fitness and virulence is crucial for improved prevention and treatment. *Campylobacter* faces challenges upon colonization in a host; it has to find itself a niche in a hostile environment and at the same time compete with other (commensal) bacteria present. Genome sequencing of numerous strains has revealed genomic flexibility in *C. jejuni* and *C. coli* and has indicated possible host-specific genotypes but has not yet identified possible virulence determinants (Van Putten et al. 2009; Yahara et al. 2017). Similarly, genome-wide functional screening (using mutant panels) is beginning to provide evidence for fitness, colonisation and virulence determinants besides flagella and glycosylation systems (de Vries et al. 2017a, b). To date, *C. jejuni* has been subject to metabolomics studies where novel glycosylation events of flagella were observed (McNally et al. 2007, 2006; Logan et al. 2009), resistance to antibiotic (Li et al. 2015), metabolic mutant differentiation (Howlett et al. 2014), and preferential utilisation of amino acids was shown through NMR analysis of depletion from complex growth medium (Wright et al. 2009).

Our study represents the first global (untargeted) metabolomics analysis of the extracellular metabolome of *C. jejuni* and its response to the presence of excess supplementary carbon sources. Although untargeted metabolomics studies have the technical limitation of not providing absolute quantifications for the measured metabolites, increasing the spectrum of small molecules reported could provide additional valuable insights into novel compounds with potential roles in microbe–microbe and microbe–host interactions. Therefore, we assessed the impact of either glutamate or fucose supplementation of growth medium on the *C. jejuni* strain 11168 phenotype by its substrate usage and extracellular metabolome as readouts.

**Fig. 5:**
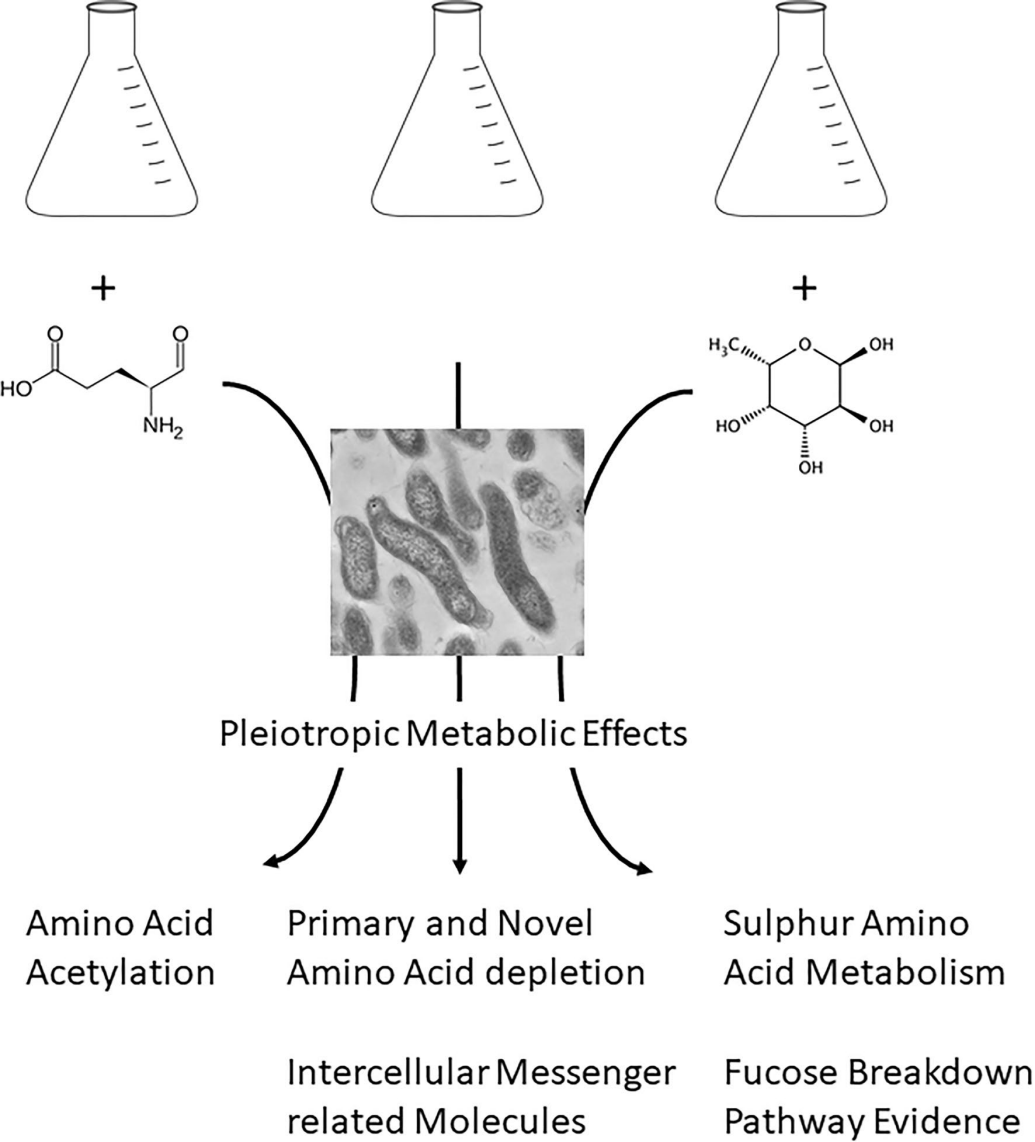
Schematic overview of study: one bacterial strain under three conditions revealed pleiotropic metabolic effects upon growth whereas two different supplementations result in specific metabolic characteristics

Our findings show that *Campylobacter* produces a large variety of metabolites even without supplementation and that the added substrates cause increased and enriched secretion of small molecules (Fig. 5). Our treatments may reflect events consequent to ‘local enrichment’ of fucose or glutamic acid—arising through host, gut co-colonizers or dietary events—as a result of which *Campylobacter* alters metabolite production and export thus influencing its impact on competing bacteria as well as host function. For example, mercaptomethylglutaric acid was found to be released in higher abundancies upon both glutamic acid and fucose supplementation. It has been reported that this metabolite inhibits gamma-glutamyl hydrolase (Whitehead et al. 1987), an enzyme involved in the metabolism of folates.

We confirmed substrates as serine, proline, aspartate, and glutamate, as well as glutamine (all used as carbon sources) were depleted from the MEMα medium. Additionally, methionine and cystine were depleted from the medium supporting a previous study indicating methionine auxotrophy among Campylobacters and use of Cys as sulphur source (Alazzam et al. 2011; Tenover and Patton 1987; Vorwerk et al. 2014). To this we add the novel observation that phenylalanine was also depleted. This observation suggests that *Campylobacter* may have additional metabolic capabilities and utilise additional substrates although it remains to be determined precisely what metabolic process(es) are involved.

This indicates that the sensing, transport, and breakdown pathways for those amino acids may play significant roles in metabolism of some strains which may contribute to occupation of specific niches. We could also observe that those amino acids were depleted even when surplus of Glu or Fuc were supplied and were entirely depleted by 4 h. The equivalent depletion of these amino acids challenges the idea of sequential utilisation of substrates as suggested by other studies (Wright et al. 2009), but would suggest a more simultaneous and dynamic use of carbon sources. Indeed, it supports the hypothesis that *Campylobacter* “senses” the nutritional compositions of the (gut) environment and adapts its metabolism in response to that (Reuter and Vliet 2013).

Over time, *Campylobacter* not only depleted amino acids and other constituents from the medium, but also excreted a range of metabolites into the medium (Fig. 2). Comparison of the Log^2^ fold changes of the medium without and with supplementation shows that these changes could be considered as ‘core extracellular metabolome’ produced by providing the essential compounds for growth. Notably, a number of metabolites previously described to be involved in chemical signalling (LuxS pathway metabolites and purine metabolites) were among those with the largest fold-change (Fig. 2), suggesting increased activity of this pathway which is associated with virulence (Plummer 2012). Both glutamate and fucose supplementation promote the growth of this strain to similar extents yet the effects on metabolome are profoundly distinct. Upon glutamic acid supplementation (Fig. 3), we mainly observe ‘enhanced production’ of metabolites, in particular a variety of acetyl-amino acids. Amongst others, the aforementioned Mercaptomethylglutaric Acid was annotated, as well as *N*-acetylethanolamine which is another potential signalling molecule released upon glutamate acid supplementation.

Upon addition of fucose, an even greater number of metabolites are released into the extracellular metabolome with sulphur-containing metabolites being dominant (Fig. 4). The global changes we observe in the extracellular metabolome reflect those measured by microarray, i.e. not specific to fucose uptake and utilisation pathway (Stahl et al. 2011; Alghafari 2016). Thus, the presence and use of fucose has major influence on physiology of *C. jejuni* strain 11168—and presumably on other fucose locus-positive strains that predominate in human infections—by reprogramming its metabolism. This interesting area warrants further investigation as to if and how these metabolites are bioactive in the gut environment.

Notably, the metabolic pathway by which fucose is utilised in *C. jejuni* has not been fully elucidated. A previous study (Stahl et al. 2011) identified potential similarity to the fucose pathway of *Xanthomonas campestris* pv. *campestris*; however, this was not confirmed via detection of metabolites or by demonstration of orthologues of the genes encoding fucose utilisation enzymes hence remained speculative. Our metabolomics analysis has added evidence specifying fucose utilisation in *C. jejuni* by recognizing intermediates in the fucose breakdown pathway such as Fuconolactone and Fuconate. Further functional prediction of both *X. campestris* and *C. jejuni* fucose locus proteins identified 4 potential functional orthologues in the latter although further investigation will be necessary to verify their activities (see Supplementary Table S2).

The global metabolomic profiling approach based on pHILIC-MS combined with MS/MS experiments as aid in structural elucidation of metabolites by spectral matching, in-silico predictions (Ridder et al. 2013) and molecular networking (Wang et al. 2016) has proven to be a successful approach to gain additional insights in bacterial phenotypes. Note that 23 out of the 101 metabolites presented in this paper could be matched to a standard (MSI MI level 1), but another 39 could be matched to a standard or other candidates could be eliminated based on diagnostic structural evidence (MSI MI level 2), and another 27 could be tentatively identified or classified based on their MS/MS patterns (MSI MI level 3), leaving only 12 at MSI MI level 4, as metabolic features consistently present across a number of samples. This underlines the enhanced metabolite annotation power that MS/MS experiments can provide. Very recent developments that group metabolites based on shared substructures discovered in and learnt from MS/MS spectra may further increase the annotation power and efficiency underlining the importance of obtaining fragmentation data of complex biological extracts full of primary and specialized molecules of which the majority is uncharacterized (van der Hooft et al. 2016b, 2017).

Our observations on substrate usage together with the annotation and identification of 101 metabolites in the *C. jejuni* extracellular metabolome preparations indicate that provision of this organism with different substrates results in substantive alterations to the extracellular metabolome suggesting that reprogramming of metabolism is not focussed on pathways utilising single substrates but, rather, is occurring widely in response to nutrient sources. Our study also highlights the importance of good chemical analysis of the starting conditions for in vitro studies, as addition of one single compound (fucose) can trigger the production of several novel compounds that could play a role as signalling molecule and/or carbon source. Finally, our findings also show that *Campylobacter* does not have ‘one metabolome’, but a core metabolome and a rather adaptive metabolome that it changes according to the presence of external factors (Fig. 5). Gaining the capability for absolute quantification of the measured metabolites in future will help assess their potential biological role.

This study indicates that the composition of substrates available to *C. jejuni* in its environment has a major influence on its physiology which could substantially influence colonisation and pathogenicity in this organism’s preferred niche, the intestinal tracts of avian and mammalian species.

## Electronic supplementary material

Below is the link to the electronic supplementary material.

Supplementary material 1 (DOCX 405 KB)

Supplementary material 2 (XLSX 55 KB)
