# Supplementary material for: Unexpected differential metabolic responses of Campylobacter jejuni to the abundant presence of glutamate and fucose
Source: Metabolomics. 2018 Oct 23;14(11):144. doi: 10.1007/s11306-018-1438-5 (PMC6208705; doi:10.1007/s11306-018-1438-5)
Supplement: Supplementary file 1 — Supplementary material 1 (DOCX 405 KB) [file 11306_2018_1438_MOESM1_ESM.docx]

**Supplementary Information to:**

**Unexpected differential metabolic responses of *Campylobacter jejuni* to the abundant presence of glutamate and fucose**

Short title: Metabolic responses of *Campylobacter jejuni*

Justin J.J. van der Hooft^1*^

Wejdan Alghafari^2,3,4^, Eleanor Watson^3^, Paul Everest^5^,

Fraser R. Morton^1^, Karl E.V. Burgess^1^,

David G. E. Smith^3*^

^1^Glasgow Polyomics, College of Medical, Veterinary and Life Sciences, University of Glasgow, Glasgow G12 8QQ, United Kingdom.

^2^King Abdulaziz University, Jeddah 21589, Kingdom of Saudi Arabia

^3^Institute of Biological Chemistry, Biophysics & Bioengineering, Heriot-Watt University, Edinburgh, EH14 4AS, United Kingdom.

^4^Moredun Research Institute, Pentlands Science Park, Bush Loan, Penicuik EH26 0PZ, United Kingdom.

^5^Institute of Infection, Immunity and Inflammation, University of Glasgow, Glasgow G12 8TA, United Kingdom.

*corresponding authors

Supplementary Table S1: Medium components of Minimum Essential Medium α (MEMα) (Gibco® 41061)

| **Components** | **Molecular Weight** | **Concentration in mg/L** | **and mM** |
| --- | --- | --- | --- |
| **Amino Acids** | | | |
| Glycine | 75.0 | 50.0 | 0.6666667 |
| L-Alanine | 89.0 | 25.0 | 0.28089887 |
| L-Arginine | 211.0 | 105.0 | 0.49763033 |
| L-Asparagine-H2O | 150.0 | 50.0 | 0.33333334 |
| L-Aspartic acid | 133.0 | 30.0 | 0.22556391 |
| L-Cysteine hydrochloride-H2O | 176.0 | 100.0 | 0.5681818 |
| L-Cystine 2HCl | 313.0 | 31.0 | 0.09904154 |
| L-Glutamic Acid | 147.0 | 75.0 | 0.5102041 |
| L-Glutamine | 146.0 | 292.0 | 2.0 |
| L-Histidine | 155.0 | 31.0 | 0.2 |
| L-Isoleucine | 131.0 | 52.4 | 0.4 |
| L-Leucine | 131.0 | 52.0 | 0.39694658 |
| L-Lysine | 183.0 | 73.0 | 0.3989071 |
| L-Methionine | 149.0 | 15.0 | 0.10067114 |
| L-Phenylalanine | 165.0 | 32.0 | 0.19393939 |
| L-Proline | 115.0 | 40.0 | 0.3478261 |
| L-Serine | 105.0 | 25.0 | 0.23809524 |
| L-Threonine | 119.0 | 48.0 | 0.40336135 |
| L-Tryptophan | 204.0 | 10.0 | 0.04901961 |
| L-Tyrosine disodium salt | 225.0 | 52.0 | 0.23111111 |
| L-Valine | 117.0 | 46.0 | 0.3931624 |
| **Vitamins** | | | |
| Ascorbic Acid | 176.0 | 50.0 | 0.2840909 |
| Biotin | 244.0 | 0.1 | 4.0983607E-4 |
| Choline chloride | 140.0 | 1.0 | 0.007142857 |
| D-Calcium pantothenate | 477.0 | 1.0 | 0.002096436 |
| Folic Acid | 441.0 | 1.0 | 0.0022675737 |
| Niacinamide | 122.0 | 1.0 | 0.008196721 |
| Pyridoxal hydrochloride | 204.0 | 1.0 | 0.004901961 |
| Riboflavin | 376.0 | 0.1 | 2.6595744E-4 |
| Thiamine hydrochloride | 337.0 | 1.0 | 0.002967359 |
| Vitamin B12 | 1355.0 | 1.36 | 0.0010036901 |
| i-Inositol | 180.0 | 2.0 | 0.011111111 |
| **Inorganic Salts** | | | |
| Calcium Chloride (CaCl2) (anhyd.) | 111.0 | 200.0 | 1.8018018 |
| Magnesium Sulfate (MgSO4) (anhyd.) | 120.0 | 97.67 | 0.8139166 |
| Potassium Chloride (KCl) | 75.0 | 400.0 | 5.3333335 |
| Sodium Bicarbonate (NaHCO3) | 84.0 | 2200.0 | 26.190475 |
| Sodium Chloride (NaCl) | 58.0 | 6800.0 | 117.24138 |
| Sodium Phosphate monobasic (NaH2PO4-H2O) | 138.0 | 140.0 | 1.0144928 |
| **Ribonucleosides** | | | |
| Adenosine | 267.0 | 10.0 | 0.037453182 |
| Cytidine | 243.0 | 10.0 | 0.041152265 |
| Guanosine | 283.0 | 10.0 | 0.03533569 |
| Uridine | 244.0 | 10.0 | 0.040983606 |
| **Deoxyribonucleosides** | | | |
| 2'Deoxyadenosine | 251.0 | 10.0 | 0.03984064 |
| 2'Deoxycytidine HCl | 264.0 | 11.0 | 0.041666668 |
| 2'Deoxyguanosine | 267.0 | 10.0 | 0.037453182 |
| Thymidine | 242.0 | 10.0 | 0.041322313 |
| **Other Components** | | | |
| D-Glucose (Dextrose) | 180.0 | 1000.0 | 5.5555553 |
| Lipoic Acid | 206.0 | 0.2 | 9.708738E-4 |
| Sodium Pyruvate | 110.0 | 110.0 | 1.0 |


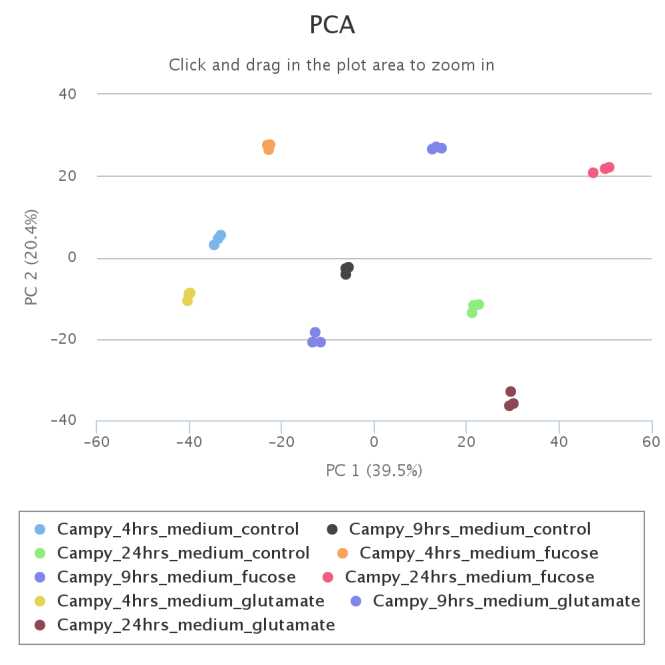


Figure S1 - Principal component analysis (PCA) reveals different metabolite profiles for each condition and time point with tight grouping for triplicate experiments.

Figure S2 - Amino acid analysis using IDEOM identifications. Fold change ratios (mean of triplicate measurements – error bars represent standard deviation up and down) of amino acid peaks of 24 Hrs over 0 Hrs samples for medium only (Med, medium with added Fucose (Fuc), and medium with added Glutamic Acid (Glu). It can be seen that several amino acids are preferably depleted from the medium. Further inspection revealed that Aspartic Acid, Glutamine, Methionine, Proline, and Serine are all completely depleted from the medium within 4 Hrs. Glutamic Acid was depleted under all conditions; in the Glutamic Acid added condition the ratio change is less pronounced due to the Glutamic Acid surplus abundance.

Figure S3 – Ratio of metabolite intensities of 0 hrs over 24 hrs growth of 8 potential Carbon sources for *Campylobacter*. Note how Ascorbic Acid and Nicotinamide are completely depleted for all conditions; however, Pyruvate is much less depleted upon addition of Fucose to the medium.

Figure S4 – Metabolites depleted from medium as found by differential metabolites in 4 hrs and 24 hrs growth in medium only conditions. It can be seen that most metabolites are part of a common set of metabolites used by *C. jejuni* upon growth. Please note that Glutamic Acid is used upon addition of excess of Glutamic Acid; however, due to its high initial abundance, the fold change is nearly undetectable.

**Supplementary Section 1 – Core extracellular metabolome of *Campylobacter jejuni***

5-Hydroxyindoleacetate was also found in *Campylobacter’s* extracellular metabolome. This is an indole-containing Tryptophan derived metabolite with potential roles in signalling or microbe-host interactions, given the number of indole-containing metabolites that possess hormonal functions^1^. The (increase in) Uracil excretion is as other purine metabolites associated with pathogeny and chronic inflammation^2^.

**Supplementary Section 2 – *Campylobacter jejuni’s* extracellular metabolome in presence of excess Glutamic Acid**

The presence of Glutamic Acid in the growth medium caused an increase in secretion of acetylated amino acids into the medium as compared to normal growth medium (see Figure 2 and Supporting Table 2). These acetylated amino acids could be signalling molecules or breakdown products of acetylated bacterial proteins^3^. Another option is that glutamate breakdown (see below) results in excess acetate that *Campylobacter* needs to get rid of – the acetylated amino acids (and other acetylated products) could be formed biologically or chemically within the cell, or chemically outside the cell; our study is not able to differentiate between those options.

The (ten-fold increased as compared to normal growth medium) secretion of mercaptomethylglutaric acid is interesting as it is described to inhibit gamma-glutamyl hydrolases^4^. This finding is interesting because this metabolite could form part of the virulence of *Campylobacter jejuni* and its increased secretion, also upon Fucose addition, may in part explain the advantage *C. jejuni* obtains when these substrates are present.

The secretion of 2-hydroxy-glutaric acid could be explained by the metabolite being part of a potential glutamate breakdown pathway as proposed in^5^. Increased availability of Glutamic Acid would then result in increased production of acetate – which may be the source of the acetylated species we observe. That would also add evidence to the Glutamic Acid breakdown pathway with 2-Ketoglutaric Acid and 2-Hydroxyglutaric Acid as intermediates and acetate and butyrate as end products. Further inspection did not yield evidence for the presence of 2-keto-glutaric acid and butyric acid, but this does not necessarily mean that they are not produced – other analytical techniques might be able to detect those if indeed they are produced and released.

**Supplementary Section 3 – *Campylobacter jejuni’s* extracellular metabolome in presence of excess Fucos****e**

# SUPPLEMENTARY TABLE S2 - Comparison of functional motifs of fucose locus proteins sequences from *X. campestris* pv. *campestris* ATCC 33913 and *C. jejuni* subsp. *jejuni* NCTC 11168 – ATCC 700819

| **Metabolite** | **Protein in *Xanthomonas* fucose operon** | **IPR & PFAM designation(s)** | **Potential match in *C. jejuni* 11168 fucose locus** | **IPR & PFAM designations** |  |
| --- | --- | --- | --- | --- | --- |
| Fucose (extracellular) |  |  |  |  |  |
| ↓ | Xcc4071 | IPR011701 - Major facilitator superfamily  IPR005275 - L-fucose permease FucP  IPR020846 - Major facilitator superfamily domain  PF07690 (MFS_1) | Cj0486 (*fucP*) | IPR011701 - Major facilitator superfamily  IPR005275 - L-fucose permease FucP  IPR020846 - Major facilitator superfamily domain  PF07690 (MFS_1) |  |
| Fucose (intracellular) |  |  |  |  |  |
| ↓↑ |  |  |  |  |  |
| L-fuco-α-pyranose |  |  |  |  |  |
| ↓↑ | Xcc4070 | IPR008000 - Rhamnose/fucose mutarotase  PR011008 - Dimeric alpha-beta barrel  PF05336 (rhaM) | Cj0488 | IPR008000 - Rhamnose/fucose mutarotase  IPR011008 - Dimeric alpha-beta barrel  PF05336 (rhaM) |  |
| L-fuco-β-pyranose |  |  |  |  |  |
| ↓ | Xcc4065 | IPR023210 - NADP-dependent oxidoreductase domain  PF00248 | Not identified |  |  |
| **L-fuconolactone** |  |  |  |  |  |
| ↓ | Xcc4066 | IPR032466 - metal-dependent hydrolase  IPR006680 - amidohydrolase  PF04909 | Cj0487 | IPR032466 - Metal-dependent hydrolase  IPR006680 - Amidohydrolase-related  PF04909 (Amidohydro_2) |  |
| **L-fuconate** |  |  |  |  |  |
| ↓ | Xcc4069 | IPR034610 - L-fuconate dehydratase  IPR029017 - Enolase N-terminal domain-like  IPR013341 - Mandelate racemase/muconate lactonizing enzyme, N-terminal domain  IPR029065 - Enolase C-terminal domain-like  IPR013342Mandelate racemase/muconate lactonizing enzyme, C-terminal  PF02746 (MR_MLE_N)  PF13378 (MR_MLE_C) | Not identified |  |  |
| **2-keto-3-deoxy-L-fuconate** |  |  |  |  |  |
| ↓ | Xcc4067 | IPR002347 - Short-chain dehydrogenase/reductase SDR  IPR016040 - NAD(P)-binding domain  IPR020904 - Short-chain dehydrogenase/reductase, conserved site  PF13561 (adh_short_C2) | Cj0485 | IPR002347 - Short-chain dehydrogenase/reductase SDR  IPR016040 - NAD(P)-binding domain  PF13561 (adh_short_C2) |  |
| 2,3-diketo-3-deoxy-L-fuconate |  |  |  |  |  |
| ↓ | Xcc4068 | IPR011234 - Fumarylacetoacetase, C-terminal-related;  PF01557 (FAA_hydrolase) | Not identified |  |  |
| L-lactate + **pyruvate** |  |  |  |  |  |
|  |  |  |  |  |  |


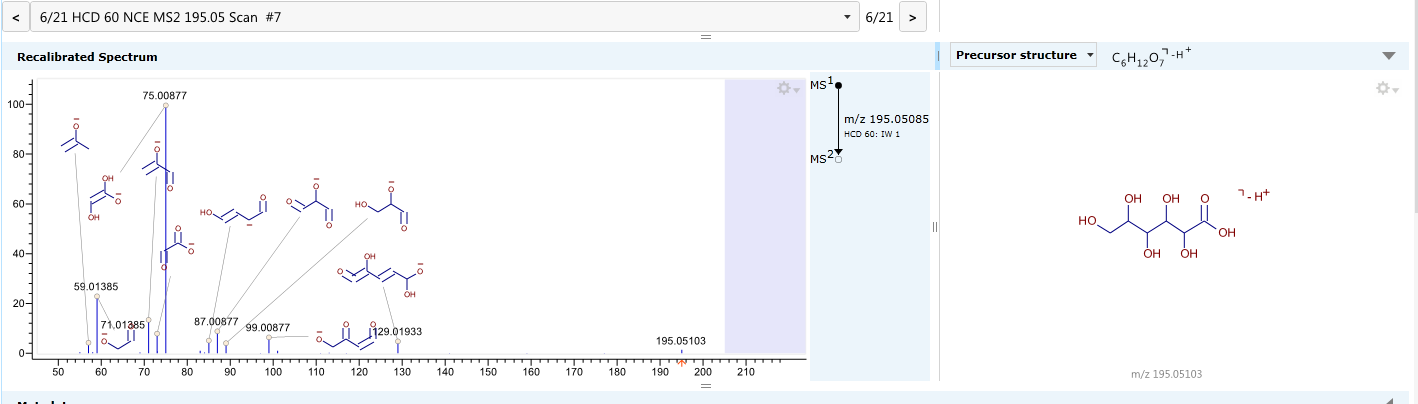

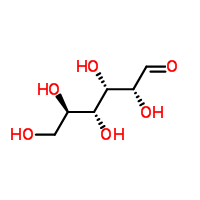

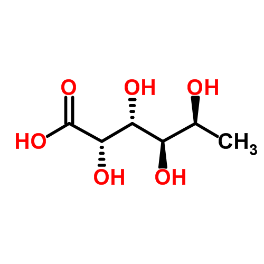

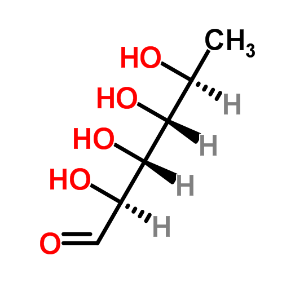
Figure S-5 – Metabolite Annotation of Fuconate by MS/MS pattern comparisons to fragmentation of Gluconate. The C_2_H_3_O_3_ [M-H]^-^ fragment is a diagnostic mass fragment containing the carboxylic acid group – the part of Fuconate and Gluconate that are the same.

MzCloud.org

Gluconate

Gluconate

Glucose (isomer of Fuconate)

Glucose (isomer of Fuconate)

L-Fuconate

L-Fuconate

L-Fucose

L-Fucose

Gluconate

Gluconate

MzCloud.org

Glucose

(isomer of

Fuconate)

The ability of certain *C. jejuni* strains to utilise fucose has been described although evidence for cognate enzymes and reaction intermediaries has been incomplete. Through this investigation, several compounds corresponding to fucose metabolism were identified. The annotation of fuconate is shown here as an example. This indicates the value of mass spectrometry-based metabolomics in annotation of metabolites and elucidating novel metabolic pathways which could lead to novel bioactive compounds.

Gluconate

Gluconate

MzCloud.org

Glucose

(isomer of

Fuconate)

The ability of certain *C. jejuni* strains to utilise fucose has been described although evidence for cognate enzymes and reaction intermediaries has been incomplete. Through this investigation, several compounds corresponding to fucose metabolism were identified. The annotation of fuconate is shown here as an example. This indicates the value of mass spectrometry-based metabolomics in annotation of metabolites and elucidating novel metabolic pathways which could lead to novel bioactive compounds.


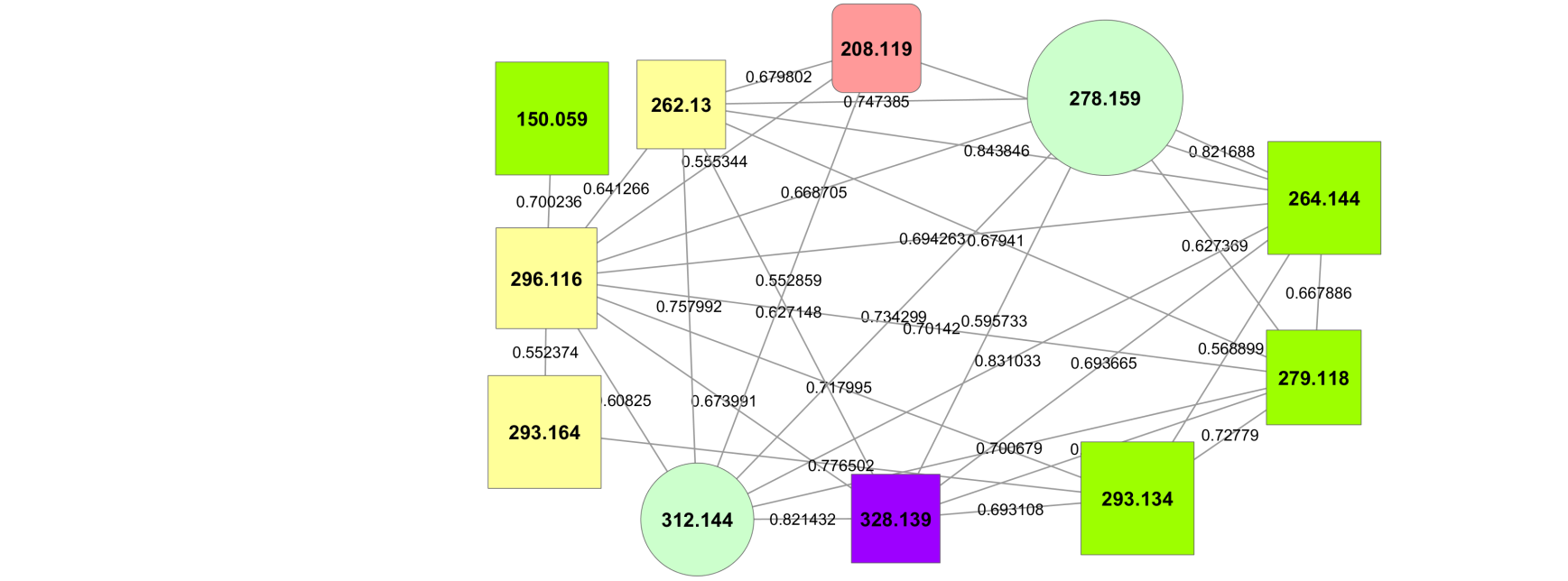


Figure S-6 - Molecular networking results – cluster of compounds around methionine and fucosylmethionine reveals a range of fucosyl-amino acids. Methionine (150.059 node) is connected to 296.116 (Fucosylmethionine), which on its turn is connected to 10 nodes all containing Fucosyl Amino Acids – with cosine scores over 0.55 due to the presence of Fucose related mass fragments: C_6_H_9_O_3_, C­_4_H_5_O_2_, C_3_H_7_O_2_, and C­_4_H_7_O (all [M+H]^+^).

References:

1 Berstad, A., Raa, J. & Valeur, J. Indole – the scent of a healthy ‘inner soil’. *Microbial Ecology in Health and Disease* **26**, 10.3402/mehd.v3426.27997, doi:10.3402/mehd.v26.27997 (2015).

2 Lee, K.-A. *et al.* Bacterial-Derived Uracil as a Modulator of Mucosal Immunity and Gut-Microbe Homeostasis in Drosophila. *Cell* **153**, 797-811, doi:<http://dx.doi.org/10.1016/j.cell.2013.04.009> (2013).

3 Hu, L. I., Lima, B. P. & Wolfe, A. J. Bacterial Protein Acetylation: the Dawning of a New Age. *Molecular microbiology* **77**, 15-21, doi:10.1111/j.1365-2958.2010.07204.x (2010).

4 Whitehead, V. M., Kalman, T. I. & Vuchich, M.-J. Inhibition of gamma-glutamyl hydrolases in human cells by 2-mercaptomethylglutaric acid. *Biochemical and Biophysical Research Communications* **144**, 292-297, doi:<http://dx.doi.org/10.1016/S0006-291X(87)80509-0> (1987).

5 Buckel, W. & Barker, H. A. Two Pathways of Glutamate Fermentation by Anaerobic Bacteria. *Journal of Bacteriology* **117**, 1248-1260 (1974).
